# Concussion knowledge among amateur motocross riders

**DOI:** 10.2217/cnc-2016-0004

**Published:** 2016-07-06

**Authors:** Kristina O Miller, Jody L Langdon, Glenn P Burdette, Thomas A Buckley

**Affiliations:** 1 Department of Intercollegiate Athletics, University of Maryland Baltimore County, Baltimore, MD 21250, USA; 2 School of Health & Kinesiology, Georgia Southern University, Statesboro, GA 30460, USA; 3 Department of Kinesiology & Applied Physiology, University of Delaware, Newark, DE 19716, USA

**Keywords:** brain, concussion reporting, extreme sports, mild traumatic brain injury

## Abstract

**Aim:**

There has been considerable increase in concussion awareness and risks; however, extreme sports such as motocross have received scant attention. The purpose of this study was to assess concussion knowledge among motocross riders and determine differences based on demographic factors.

**Methodology & results:**

782 motocross riders responded to an Internet-based questionnaire, and participant's knowledge score was 14.3 ± 2.7 out of 20 and symptom recognition was 6.8 ± 1.4 out of 8. Riders who had performed baseline concussion testing or received formal concussion education demonstrated higher knowledge scores. Rider's demographics did not predict outcome measures.

**Conclusions:**

Considerable misconceptions and lack of symptom knowledge persist among motocross riders and these results can be used for future interventions to improve concussion reporting.

## Background

There are an estimated 1.6–3.8 million sports-related concussions, which occur in the USA annually; however, this likely underestimates the true incidence as unrecognized and unreported concussions remain highly prevalent [1,2]. Sports only account for 4–11% of all traumatic brain injury (TBI) in children, whereas motor vehicle accident (61%) and bicycle crashes (20%) account for far greater incidence rates [3]. Motocross is a popular extreme sport with over 200,000 active members of the American Motorcyclist Association (AMA), which annually sponsors almost 3000 events across over 1100 charter clubs [4]. Motocross involves riding a two-wheeled vehicle (including dirt bikes, minibikes, pocket bikes, pocket rockets and minimotos) along three mile off-road tracks at speeds up to 100 miles per hour with 0–60 acceleration times of less than 5 s [5,6]. Not surprisingly, injury rates in motocross competitions are high with an estimated 22 injuries per 1000 h of motorcycle riding. Practice sessions can have higher overall injury rates than races and spinal fractures comprise 5.8% of all fractures with a third of these experiencing permanent neurological sequel [5,7,8].

Despite the large number of participants and obvious high risk associated with participation, there is limited injury epidemiology data or surveillance systems in place to track motocross injuries. In hospital-based studies, motocross is responsible for high injury severity and costs (mean = $14,947 per hospital injury) and head injuries account for 20–25% of all motocross-related hospital visits [6,9,10]. At a single race event study, up to 50% of riders reported experiencing a concussion-related symptom from riding in the previous season and 33% reported multiple incidents. Most riders (61%) continued racing that day and 24% continued the remainder of the season without interruption, however, 78% did eventually seek medical treatment for their symptoms [11]. Furthermore, almost a quarter of riders (23.7%) reported chronic headaches that started after falls or crashes [11]. Motocross does requires baseline neurocognitive testing at the professional, but not amateur, levels. Overall there is inconsistent or nonexistent formal assessment, thereby requiring participants to self-report suspected concussions incidents and symptoms to healthcare providers [11].

In school-based team sports, concussion reporting rates have increased over time and many concussion-related misconceptions have been reduced; nonetheless, reporting remains suboptimal [2,12–14]. Concussion underreporting is generally subcategorized into either ‘unrecognized’ or ‘intentionally unreported’ [2]. The intentionally unreported category involves numerous complex sociocultural considerations and is receiving considerable attention in school-based team sports [15–18]. Unrecognized concussions are those injuries that were likely concussions but were never reported to a healthcare professional, as opposed to intentionally concealing the injury, and only identified subsequently when specific symptom lists or descriptions were provided [11,19,20]. As the overwhelming majority of concussions do not include loss of consciousness or obvious overt symptoms, individual self-recognition of symptoms is critical to initiating proper healthcare and reducing the risk of subsequent injury [21,22].

Extreme sports participants, such as motocross riders, are likely culturally different (older, more experienced and overwhelmingly male) from school-based team sports and therefore previously utilized concussion educational interventions may not be transferable [4,5]. To develop effective concussion education programs for motocross, identification of current knowledge and concussion-related misconceptions (e.g., loss of consciousness is required for a concussion to occur) is the logical first step. While increasing knowledge alone is inadequate to improve reporting likelihood or intention, identification of knowledge gaps can be useful to addressing the unrecognized concussions. Therefore, the purpose of this study was to assess concussion knowledge, symptom recognition and misconceptions among a diverse pool of motocross riders. Based on clinical experience, we hypothesized that motocross participants would have poorer concussion knowledge and subscribe to more misconceptions than school-based sports participants.

## Methods

### Participants

There were a total of 1396 responses to the online recruitment, of which 904 completed the questionnaire; however, an additional 122 removed due to self-reporting to being a professional motocross rider (n = 76), being a minor (n = 13), self-reporting not being a motocross rider (n = 16), multiple responses to the questionnaire (n = 15) and likely falsified information (n = 2) based on unrealistic and/or highly improbably responses. Thus, a total of 782 individuals (age: 30.6 ± 12.0) were included. Individuals had to self-report being at least 18 years of age and currently participating in motocross as an amateur rider. Any incomplete questionnaires were excluded. More detailed demographic information is located in Table 1. The respondents provided electronic informed consent prior to participating in this study as approved by the University's Institutional Review Board.

**Table 1.** **Respondent demographics.**

**Respondent demographics**	
Age (years)	30.6 ± 12.0 (range: 18–65)

Race	White: 95.3%; Hispanic: 2.3%
	Native American: 0.6%
	Black: 0.4%; Asian: 0.4%; other/not reported: 1.0%

Gender	85.0% male (665/782)

Motocross experience (years)	12.2 ± 9.9 (range: 1–41)

Races per year	14.1 ± 12.8 (range: 1–54)

Prior concussion history	75.1% (587/782)

Number of prior concussions	3.5 ± 2.4 (range: 0–11)
	≥3 concussions: 42.4%

Received formal concussion education	24.1% (188/779)

Performed baseline computerized neuropsychological test	5.4% (42/777)

Data are presented as mean ± standard deviation and the range is provided in parenthesis below.

### Instrumentation

#### Concussion knowledge questionnaire

A 46-item Internet-based questionnaire was developed for the purpose of this study, based on contemporary concussion literature and recommendations and delivered via secure software (Qualtrics Labs Inc., UT, USA). The questionnaire consisted of four components: first, 10 demographic questions; second, 17 mostly true/false and multiple choice questions that pertained to general concussion knowledge; third, three scenarios following a possible concussion and fourth, 16-item concussion signs and symptoms recognition checklist [2,14,23]. The reliability of the concussion signs and symptoms recognition was favorable (Cronbach's alpha = 0.83). The questionnaire was initially pilot tested on 33 physically active college students to ascertain content clarity and were similar to a previous study and the reliability within the pilot testing, as calculated by the KR-20, was moderate (0.62) [14,23]. Content and face validity was also established through piloting the questionnaire with professional motocross riders as well as experienced concussion healthcare providers and revisions were made based on the feedback received.

### Procedures

Participant recruitment was primarily executed through the use of social media outlets, namely Facebook™ and Twitter™. Since the population of interest was small, the primary researcher posted a specific script with a questionnaire link on the Facebook group walls that included motocross tracks/riders/enthusiasts/sponsors and also had the administrator of these groups post it under their name as well. In addition, the primary researcher also sent specific short messages, or ‘tweets’, to motocross tracks/riders/enthusiasts/sponsors found through searches of Twitter users. Email was sent to AMA district representatives as well as motocross track guide representatives, which also followed a specific script and asked them to send the questionnaire link to all the riders within their respective districts. The questionnaire was only provided in English and was distributed to US-based organizations/pages; however, the national origin or primary language of the respondents is unknown. The first page of the questionnaire contained the informed consent. Once finished, respondents were entered into a raffle to win electronic gift cards. There is considerable support for sampling of this type in the literature, especially when working with populations that are small and/or difficult to recruit otherwise. Social media recruitment has been regarded as a successful and meaningful sampling strategy among young adult cancer survivors [24], smoking cessation program participants [25] and HIV-positive individuals [26]. Among these studies, recruitment of participants was as, if not more, successful than traditional methods, but response rates cannot be provided as the denominator cannot be determined.

### Data analysis

The independent variables were identified in the first section of the questionnaire and included demographics, self-reported status of receiving formal concussion education and undergoing baseline-computerized neuropsychological testing, which were coded as categorical variables (‘yes’ or ‘no’). The number of years racing, average number of races per year and self-reported number of prior concussions were coded as continuous variables based on the number provided. The dependent variables were the 20 concussion knowledge questions in Sections 2 (17 questions) and 3 (3 scenarios) from the questionnaire and the 8 correct concussion symptoms (Section 4), thus a perfect score was 28 with a higher score reflecting better concussion knowledge.

### Statistical analysis

Frequencies and means/standard deviations were calculated to provide respondent's demographic information and to establish the scores of the dependent variables. To identify potential relationships, both Spearman and Pearson correlations were calculated, where appropriate, between all demographic variables and the total knowledge score (0–28). After ensuring that all assumptions were met and the data were normally distributed, a multiple regression was then performed for total knowledge score, using only those demographic variables that had significant relationships with the total knowledge score.

To further investigate the data and identify potential group differences, separate one-way ANOVAs were performed comparing the categorical demographic variables (formal concussion education and neuropsychological baseline testing) with the total knowledge score. Race (95.3% white) and gender (85.0% male) were not assessed due to lack of diversity in the sample, but consistent with overall motocross demographics [4]. All alpha levels were set at 0.05 with the exception of formal concussion education, which was set at 0.01 to adjust for potential nonequivalency of groups.

## Results

### Descriptive statistics

The respondents mean score on the 20 concussion knowledge questions was 14.3 ± 2.7 (median: 15, mode: 16, range: 4–20; Table 2) and the symptom recognition score was 6.8 ± 1.4 (median: 7, mode: 8, range: 0–8; Table 3) for a mean overall score of 21.1 ± 3.4 (median: 22, mode: 24, range: 8–28) out of 28. Within the scenario or multiple options questions, several misconceptions were identified (Table 4).

**Table 2.** **Concussion knowledge.**

	**True**	**False**
1. Being blacked out or LOC is required to sustain a concussion	15.0%, n = 117	*85.0%, n = 665*

2. Showing S/S of a concussion should not be allowed to return to riding that same day	*91.0%, n = 712*	9.0%, n = 70

3. Youth riders suffering a concussion are more at risk of second impact syndrome	*84.5%, n = 661*	15.5%, n = 121

4. There are no long-term effects after sustaining a concussion	4.9%, n = 38	*95.1%, n = 744*

5. Once a rider has sustained a concussion, they are at a higher risk for another concussion	*81.5%, n = 637*	18.5%, n = 145

6. A concussion requires immediate removal from a practice ride or race	*89.4%, n = 699*	10.6%, n = 83

7. Memory loss, post-traumatic amnesia, is required for a rider to sustain a concussion	14.7%, n = 115	*85.3%, n = 667*

8. A bell ringer requires immediate removal from a practice or race	*47.8%, n = 374*	52.2%, n = 408

9. A helmet will prevent concussions	13.4%, n = 105	*86.6%, n = 677*

10. Children with concussion take less time to heal than adults	27.7%, n = 217	*72.3%, n = 565*

11. A second concussion will heal faster than the first concussion	3.8%, n = 30	*96.2%, n = 752*

12. A rider is still at risk of suffering a second concussion even 10 days after the concussion	*88.7%, n = 94*	11.3%, n = 88

13. Once a rider has suffered a concussion, it is important to keep them awake	91.9%, n = 719	*8.1%, n = 63*

14. A rider can get a bell ringer in the first moto and be okay to continue riding as long as they rest before the second moto	36.3%, n = 284	*63.7%, n = 498*

15. A physician or healthcare provider should evaluate a ‘bell ringer’	*59.5%, n = 465*	40.5%, n = 317

Correct answers are italicized.

**Table 3.** **Participant responses on concussion symptoms and distractors.**

**Symptom (% correctly identified)**	**Current study**	**Saunders *et al*. (2011)**	**McLeod *et al*. (2013)**	**Williams *et al*. (2015)**	**Gourley *et al*. (2010)**
	**n = 782**	**n = 150**	**n = 156**	**n = 25**	**Athlete, n = 73/parent, n = 100**

**Population**	**Motocross riders (%)**	**Coaching students (%)**	**Youth coaches (%)**	**Professional soccer players (%)**	**Youth athletes and parents (%)**
Black eye	78.3	90.0	79.5	88.0	N/A

*Blacked out/LOC*	*91.9*	*90.7*	*80.1*	*80.0*	*68.5/81.0*

*Blurred vision*	*92.5*	*93.3*	*53.8*	*92.0*	*69.9/86.0*

Chest pain	93.2	91.3	88.5	100.0	63.0/56.0

*Confusion*	*93.2*	*94.0*	*89.1*	*92.0*	*69.9/88.0*

*Dizziness*	*94.8*	*94.7*	*88.5*	*92.0*	*75.3/88.0*

Feeling sick/nausea	*80.4*	*71.3*	*55.8*	*64.0*	*50.7/82.0*

*Headache*	*94.9*	*96.7*	*77.6*	*100.0*	*78.1/87.0*

Loss of memory/amnesia	*93.1*	*64.7*	*60.3*	*52.0*	*72.6/82.0*

Nosebleed	66.8	70.7	95.5	84.0	47.9/26.0

Numbness/tingling in the arms or hands	66.2	49.3	82.7	92.0	38.4/27.0

Sharp burning pain in the neck	74.2	64.0	89.7	96.0	47.9/20.0

*Trouble sleeping*	*35.7*	*55.3*	*12.8*	*48.0*	*35.6/56.0*

Unusual sense of smell	68.0	74.7	5.8	92.0	49.3/32.0

Unusual sense of taste	69.2	75.3	7.1	100.0	50.7/34.0

Weak feeling when moving your neck	51.9	57.3	10.9	56.0	31.5/20.0

The correct answers are italicized. Participants correctly identified 6.8 ± 1.4 (median: 7, mode: 8) of the actual concussion symptoms.

**Table 4.** **Concussion-related scenarios and multiple option answers.**

	**Yes**	**No**
Scenario 1. A motocross rider receives a direct blow to the side of the head from another rider and falls to the ground. As they get up, they are dizzy and have a headache. Should the rider continue riding?	16.2%, n = 127	83.8%, n = 655

Scenario 2. A motocross rider falls off their bike and hits their head during their first moto. After going back to their trailer, they have no headache and remember everything, but they have a nosebleed and a black eye. Can the rider continue riding that day?	35.2%, n = 275	64.8%, n = 507

Scenario 3. A motocross rider receives a hit to the head during a practice ride. As the rider is checked out, it is found that they are awake, have no memory loss and feel fine at rest. When asked to ride their BMX bike, they have a mild headache. Should the rider return to riding?	30.1%, n = 235	69.9%, n = 547

	≤6 days	≥7 days

Fill in the blank: When a rider has suffered a concussion, how many days should they wait before returning to riding?	30.1%, n = 235	69.9%, n = 547

Which of the following injuries is most severe:	1–4	5. Same

1. Having your bell rung, 2. Sustaining a ding, 3. Sustaining a concussion, 4. Sustaining a mild traumatic brain injury, 5. They are all the same.	60.0%, n = 469	40.0%, n = 313

### Group comparisons

Respondents who reported taking a baseline computerized neuropsychological test had a significantly higher knowledge score than those who did not (22.3 ± 2.7 and 21.0 ± 3.5, respectively, F(1,777) = 6.1, p < 0.001, *d* = 0.43). Respondents who reported receiving formal concussion education had a significantly higher knowledge score than those who did not (21.9 ± 3.2 and 20.8 ± 3.5, respectively, F(1,777) = 14.0, p < 0.001, *d* = 0.32).

### Predicting total concussion knowledge

There were weak significant correlations between the 28 point total knowledge score and gender (r = 0.14, p < 0.001), taking a baseline neuropsychological test (r = 0.09, p = 0.014), formal concussion education (r = 0.14, p < 0.01), number of years racing (r = 0.08, p = 0.022) and age (r = 0.11, p = 0.01). Results from the multiple regression using these variables were significant (F (1,777) = 16.82, p < 0.001), accounting for 7.6% of the variance in the knowledge score (Table 5). All variables were found to be significant contributors except number of years racing.

**Table 5.** **Beta coefficients predicting total knowledge score.**

**Variable**	**B**	**SE(B)**	**β**	**t**	**Sig. (p)**
Age	0.035	0.01	0.12	3.50	<0.001

Gender	1.39	0.34	0.14	4.15	<0.001

Baseline neuropsychological test	-1.21	0.25	-0.17	-4.84	<0.001

Formal concussion education	-1.07	0.28	-0.17	-0.13	<0.001

## Discussion

Despite the wide spread popularity, high incidence of traumatic injuries including concussions and the lack of medical personnel available, motocross has received limited attention in the sports medicine literature. The main finding of this study was moderate to good concussion knowledge and symptom recognition, which was similar to earlier studies in team sports environments, but important misconceptions persist. Furthermore, there were weak positive correlations, wherein motocross riders who had taken a baseline neuropsychological tests or had received formal concussion education performed better on the concussion questionnaire. By identifying specific areas where knowledge is lacking within this population, the results of this study can be utilized to develop educational interventions, which may serve to improve symptom reporting to healthcare professionals and thereby potentially reduce both acute and long-term injury risk.

The respondent's herein demonstrated similar knowledge and misconceptions as athletes and coaches in more traditional team-based sports, albeit the timing of the published studies likely plays a role as concussions have permeated the media and public in recent years [20,23,27,28]. Overall, the respondents endorsed few misconceptions with most of the general knowledge questions exceeding 80% accuracy (Table 2). Generally, the most considerable misconceptions revolved around misunderstandings of concussion versus ‘bell ringer’ terminology along with the associated participation and medical management (Table 2). The respondents herein also showed high symptom recognition with greater than 90% recognition of six common concussion symptoms. The total number of correctly identified symptoms (6.8 ± 1.4) was similar to studies of professional soccer/football players (6.2 ± 1.5) and coaching education students (6.6 ± 1.4) [20,23]. The low awareness of difficulty sleeping, while problematic, is also the only nonacute symptom and therefore would likely not play a role in same-day participation decisions. One area of noted improvement in this study was an increased recognition of ‘memory loss/amnesia’ (93.1%) compared with earlier reports (52.0–82.0%) [14,20,23,28]. It should be noted that the term used herein was the combined ‘memory loss/amnesia’, whereas earlier studies had simply used the term ‘amnesia’ and pilot testing indicated a lack of understanding of the term amnesia. These results suggest that motocross riders are highly aware of common concussion symptoms, but it is unknown if this translates to reporting these symptoms to healthcare providers. When presented with scenarios which represented likely concussions (scenarios 1 and 3), respondents indicated a conservative approach and indicated they should not return to riding. Interestingly, professional soccer/football athletes gave similar answers on a pen and paper questionnaire, but during interviews indicated they would likely continuing participating if they viewed the match as important or that their team needed them to continue playing [20].

Respondents who indicated that they had received formal concussion education scored ˜1 point higher overall than those who did not report receiving formal concussion education; however, the effect size was moderate. The specific format of concussion education was not described herein and passive forms of educational interventions are often ineffective [30]. Furthermore, the administration of educational interventions can be challenging even in organized school-based team sports, whereas motocross provides the additional challenge of individual participation without a centralized school-based administration [31]. Providing passive educational interventions will likely increase concussion knowledge, but is unlikely to produce long-term behavioral changes [30,32]. Rather, in order to evaluate the likelihood of the timely reporting of suspected concussions; ‘intention to report’ is likely a more valuable metric than simply knowledge [33,34]. However, theory-driven and population-appropriate concussion educational interventions are lacking, but the results herein provide context specific to the extreme sport of motocross [33].

Overall, respondents scored well on the concussion knowledge questionnaire and accurately identified many of the concussion-related symptoms; however, there were outliers who performed poorly. Almost 10% of respondents (9.7% [76/182]) scored a 10 or below on the 20-item questionnaire and 6.1% (48/782) identified four or fewer concussion symptoms. Furthermore, on average 10–15% of respondents were incorrect on most concussion knowledge questions including common concussion misconceptions of requiring loss of consciousness (15.0%) or memory loss (14.7%) for a concussion to have occurred. These results were similar or better than recent studies, but present clear evidence of persistent misconceptions [14,20,23]. More concerning, 9% of respondents indicated it was acceptable for a rider experiencing concussion-related symptoms to continue participating on the same day and the rate quadrupled (36.3%) when the question was rephrased to indicate the injury was a ‘bell-ringer’. While the term ‘bell-ringer’ has been recommended to be removed from concussion terminology for over a decade, this study is consistent with previous work indicating athletes still utilize the term and mistake it for a concussion [19,35]. As a study limitation, it is certainly possible that respondents were intentionally performing poorly on the questionnaire or misunderstanding the questions; nonetheless, the relatively high rate (6–10%) of lower performers is concerning.

It is important to note that this was an anonymous online survey and a response bias in which the participant provides the answer perceived as correct (i.e., the most socially acceptable answer or the answer the participant believes the investigators desire) must be acknowledged. Furthermore, the conditions under which the questionnaire was answered are unknown and it is possible that responses could have been influenced by the environment (e.g., spouse or relative encouraging a ‘safer’ answer) or the respondent may have misrepresented their identity. Although these are potentially significant limitations, use of social media recruitment procedures have been well documented in the healthcare literature as a viable method of collecting data [24–26]. Recruiting through social media allowed the researchers to gain access to an understudied and high-risk population; however, social media recruitment is not without limitations open to potential abuse or misuse – herein, we removed two responses for providing unreasonable and/or improbably responses. Finally, a self-selection bias potentially exists whereby those individuals who had a personal history of concussion disproportionately responded to the recruitment thus skewing the results. The results of this study suggest that motocross riders have high, but incomplete, concussion-related knowledge and can accurately identify most concussion-related symptoms. Future research should consider how well this level of knowledge translates to actual reporting in this population.

## Future perspective

The topic of sports-related concussion will likely to continue to expand exponentially as the later life neuropathological consequences of concussions and repeated head trauma are elucidated. Furthermore, these findings and the extensive media coverage may fundamentally alter collision sports. Currently, extreme sports have received limited attention; however, the recent suicide of BMX rider Dave Mirra has brought new focus to the topic of head injury in extreme sports. Thus, in the next 5–10 years, it is likely extreme sports will be pressured and/or required to address the issue of head trauma by mandating baseline testing for amateur riders, improved healthcare at events, as well as new rules and regulations regarding restricted participation following a suspected concussion.

**Executive summary**

- This study evaluated the concussion knowledge and symptom recognition of amateur motocross riders.
- There were 782 adult amateur motocross riders who completed the anonymous online questionnaire.
- Out of 20 knowledge-based questions, the respondents correctly answered a mean of 14.3 ± 2.7, the median was 15 and the range was from 4 to 20.
- Of the eight concussion-related symptoms, respondents correctly identified 6.8 ± 1.4 symptoms, the mean was 7 and the range was 0 to 8.
- Respondents who self-reported prior concussion knowledge or having taken a baseline-computerized neuropsychological test scored higher than those respondents who did not self-report either.
- There were weak positive correlations between combined knowledge and symptoms (28 possible points) and gender, taking a baseline neuropsychological test, formal concussion education, number of years racing and age.
- The combined predictive model accounted for 7.6% of the variance of the model.
- Amateur motocross riders have high, but incomplete, knowledge of concussions and recognize most concussion-related symptoms.
- The most common misconceptions were related to concussion terminology and believing continued participation was acceptable on the day of injury.
- This study can provide the knowledge foundation to develop educational interventions for this unique sport population.
